# Cardio-Immunology of Myocarditis: Focus on Immune Mechanisms and Treatment Options

**DOI:** 10.3389/fcvm.2019.00048

**Published:** 2019-04-12

**Authors:** Bernhard Maisch

**Affiliations:** Faculty of Medicine, and Heart and Vessel Center, Philipps-University, Marburg, Germany

**Keywords:** myocarditis, endomyocardial biopsy, immunohistology, PCR of cardiotropic viruses, immunopathogenesis, ivIg, immunosuppressive therapy

## Abstract

Myocarditis and inflammatory cardiomyopathy are syndromes, not aetiological disease entities. From animal models of cardiac inflammation we have detailed insight of the strain specific immune reactions based on the genetic background of the animal and the infectiosity of the virus. Innate and adaptive immunity also react in man. An aetiological diagnosis of a viral vs. a non-viral cause is possible by endomyocardial biopsy with histology, immunohistology and PCR for microbial agents. This review deals with the different etiologies of myocarditis and inflammatory cardiomyopathy on the basis of the genetic background and the predisposition for inflammation. It analyses the epidemiologic shift in cardiotropic viral agents in the last 30 years. Based on the understanding of the interaction between infection and the players of the innate and adaptive immune system it summarizes pathogenetic phases and clinical faces of myocarditis. It gives an up-to-date information on specific treatment options beyond symptomatic heart failure and antiarrhythmic therapy. Although inflammation can resolve spontaneously, specific treatment directed to the causative etiology is often required. For fulminant, acute, and chronic autoreactive myocarditis without viral persistence immunosuppressive treatment can be life-saving, for viral inflammatory cardiomyopathy ivIg treatment can resolve inflammation and often eradicate the virus.

## Introduction

More than a century ago, when coronary artery disease was neglectable, inflammation of the heart (= myocarditis) was thought to be the dominant cause of any cardiac disease (1). It has been known for decades that the heart is target of immunological effector organs, the T- and B-cells, their products such as circulating antibodies, mediators and cytokines (2). Nowadays, the heart is also considered an immunological organ reacting to damage and stress (3) and even with an antibody response to stress proteins (4). This occurs on the basis of a genetic background (5) and also epigenetic mechanisms (6), which led to the distinction between familial and non-familial cardiomyopathies in the latest classification of cardiomyopathies (7) and clarification of terms in the recent position statement of the working group on myocardial and pericardial diseases (8). Further insight in treatment options has been given in 2012 (9) and recently updated in 2018 (10).

The term cardiomyopathies is much younger than myocarditis and was first used by Hickie and Hall 1960 (11). The WHO/ISFC (World Health Organization/ International Society and Federation of Cardiology) Task Force defined it as “heart muscle diseases of unknown cause” (12). In 1996 the term cardiomyopathies was applied to all heart muscle diseases, which lead to functional impairment of the heart (13). Dilated cardiomyopathy (DCM) was one of 3 main clinical phenotypes (dilated, hypertrophic, restrictive cardiomyopathy). Remarkably, the 1996 task force included inflammatory heart muscle diseases (myocarditis, perimyocarditis), hypertensive, and ischemic cardiomyopathies and other forms of heart failure of known origin in the group of secondary cardiomyopathies. Inflammatory cardiomyopathy was then defined as inflamed myocardium assessed histologically as myocarditis in association with cardiac dysfunction. The pathohistological criteria at that time were the Dallas criteria (14), which distinguished active, recurrent, healing and borderline myocarditis. The etiology was assumed to be either Infectious, toxic or autoimmune. Non inflammatory viral cardiomyopathy was defined as viral persistence in a dilated heart without ongoing inflammation. Inflammatory cardiomyopathy was further specified in a World Heart Federation consensus meeting in 1999 by quantitative immunohistological criteria for inflammation (> 14 infiltrating cells/mm^2^) (15, 16) and referred to it in the consensus document 2013 (8). These infiltrating cells in the myocardium could be T- and B lymphocytes, their activated forms and up to 4 monocytes or macrophages/mm^2^. The underlying microbial agent had to be assessed or excluded by molecular biological methods, e.g., polymerase chain reaction (PCR) or *in situ* hybridization (17).

## Lessons Learned from Animal Studies

**Figure 1:**
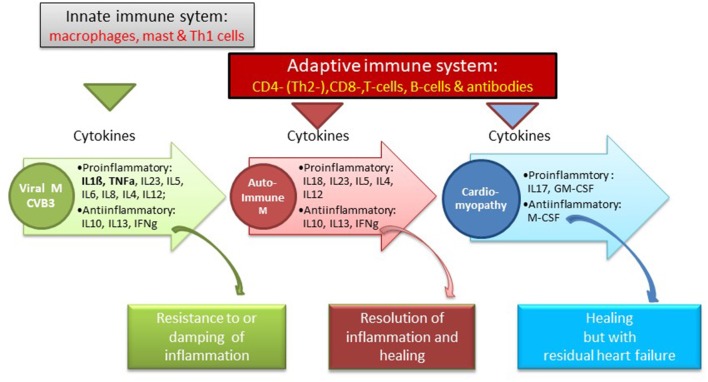
Immune system and cytokine pattern in various susceptical and resistant mouse myocarditis models [Balbc, A/J mice, after CVB3 infection with a consecutive autoimmune reaction to cardiac myosi (25)].

Animal studies have contributed much to our understanding of the role of the immune system in cardiac homeostasis and disease (18):

1) In *healthy mice hearts* all major leukocyte classes including mononuclear phagocytes, neutrophils, B and T cells are present. They can be resident or from circulating blood. The normal mouse heart also contains resident cardiac dendritic cells and mast cells.
2) Between individual cardiomyocytes a network of resident macrophages exists, which are heterogeneous and ontogenetically diverse.
3) Leukocyte distribution is not uniform but the cells adhere to niches: Embryonically derived macrophages are adjacent to coronary vasculature, fetally derived monocytes are close to endocardial trabeculae, the aortic valve is rich in dendritic cells, the AV node contains a relatively high concentration of macrophages.
4) Immune cells and macrophages in particular also participate in organ development and steady-state physiology of tissue such as housekeeping tasks for maintaining cardiac function, cell and matrix turnover, and angiogenesis.
5) Macrophages interact with the conduction system. Depletion of macrophages in mice hearts may lead to conduction abnormalities (19).
6) The pericardial adipose tissue can readily supply leukocytes during myocardial injury. Mast cells accumulate preferably in white adipose tissue.
7) It has been shown that in *injured hearts* of mice and men resident and circulating leukocytes can be activated any form of injury such as in infarction (20) or after cardiac surgery (21).
8) Genetic studies indicated that susceptibility to Coxsackievirus (CV) B3 depended on the strain of mice used for infection. Virological studies revealed that different strains of CVB developed different magnitudes of cardiac organ involvement from very active forms of myocarditis to no inflammation at all. Also organ specificity for either heart and/or pancreas depended on the susceptibility of the mice. But also infectiosity of different CVB strains was important. More recently CVB3 strains were isolated with 5-terminal deletions in genomic RNAs from a patient with idiopathic dilated cardiomyopathy. These deletions lacked portions of the 5′stem-loop I, which is a RNA secondary structure required for viral RNA replication. These findings suggest that even mutant viruses can be responsible for persistent infection. And in this changed structure they may also escape conventional PCR detection (22).
9) In the beginning of myocarditis viral infection of the heart is recognized by pattern recognition receptors (PRRs) such as toll like receptors (TLR) 2, 3, 4, 7, and 8. The downstream effects of each TLR activation may be different to each TLR, but all share a common a pro-inflammatory response. For instance after TLR2 stimulation by a damaged self-protein such as cardiac myosin monocytes produced pro-inflammatory cytokines such as IL-6, IL-8, and TNF-α. Cardiac myosin has often been used as antigen for immunization and initiation of experimental autoimmune myocarditis models. Viral murine myocarditis has focused on CVB3 infection. Signaling of the adaptor protein Myd88 downstream of TLR4 led to activation of NF-κB, which decreased survival. In contrast signaling of adaptor protein TRIF up-regulates the antiviral IFN-β response and improves survival.
10) In mice strains susceptible to infection with CVB3 myocardial disease occurs in 3 pathogenetic phases (infection, autoimmune reaction, dilated cardiomyopathy) with 3 symptomatic faces (malaise, inflammation, heart failure) (23, 24). In *phase 1* CVB3 enters the myocardial cells via the coxsackie/adenoviral receptor (CAR) hereby initiating first the innate and later the adaptive immune responses. Mast cells as early responders produce proinflammatory cytokines (TNF, IL-1ß, IL-r). Neutrophils and monocytes produce additional mediators such as IL-12. An increased production of interleukin-1b (IL-1b) and tumor necrosis factor-alpha during the early innate response to viral infection is a prerequisite for the induction of heart-specific autoimmune myocarditis. Its severity is determined by the number of T helper 1 (Th1) and Th2 cytokines. The Th1 pathway by interleukin-12 (IL-12) and gamma interferon (IFNgamma) is in principle proinflammatory and can lead to myocardial infiltration of the heart. It can also be down regulated by INFgamma production. The prototype Th2 cytokine is IL-4. It is frequent in severe forms of autoimmune myocarditis where eosinophils are prominent. Since it can go along with an IFNgamma increase, the disease is limited. IL-13, another Th2 cytokine, protects from infection, and reduces inflammation. Th17 cytokines also contribute to disease. The signature cytokine IL-17A is not essential for cardiac inflammation, but it is needed for the progression to heart failure (Figure 1) (25).

Myeloid differentiation primary response protein 88(MYD88) and IL-1 receptor-associated kinase 4(IRAK4) enhance myocardial inflammation by activating TNF receptor-associated factor 6(TRAF6) and nuclear factor-kappa b (NFKB). This decreases the production of antiviral type I interferons in the affected host. In *phase 2* after presentation by dendritric cells the antigen-specific T-cells are the key players of cardiac damage. They may be counteracted by Treg cells. Inflammation may thus be ended or go on chronically as autoimmune reaction. T helper cells promote the development of cardiac dilatation by stimulating cardiac fibroblasts (phase 3). The autoimmune reactivity develops because coxsackievirus shares epitopes with cardiac myosin (23, 26), which as endogenous antigen contributes to this chronic inflammation.

Cardiac myosin as antigen has been used as a prototype protein in experimental autoimmune myocarditis (EAM), as well as many others antigens, e.g., Troponin I. They all can be prototypes of a B-cell driven myocarditis.

In these animal models *phase 1*, the infection phase, was followed by an antiviral and an autoimmune reaction (*phase 2*) (23, 26). This 2nd phase can be followed by lethal cardiac decompensation in a fibrotic heart with severe myocyte loss (*phase 3*) or by the resolution of the inflammation.

In man a similar triphasic pathogenetic process was assumed. The corresponding clinical correlates are malaise, inflammation and heart failure.

**Figure 2:**
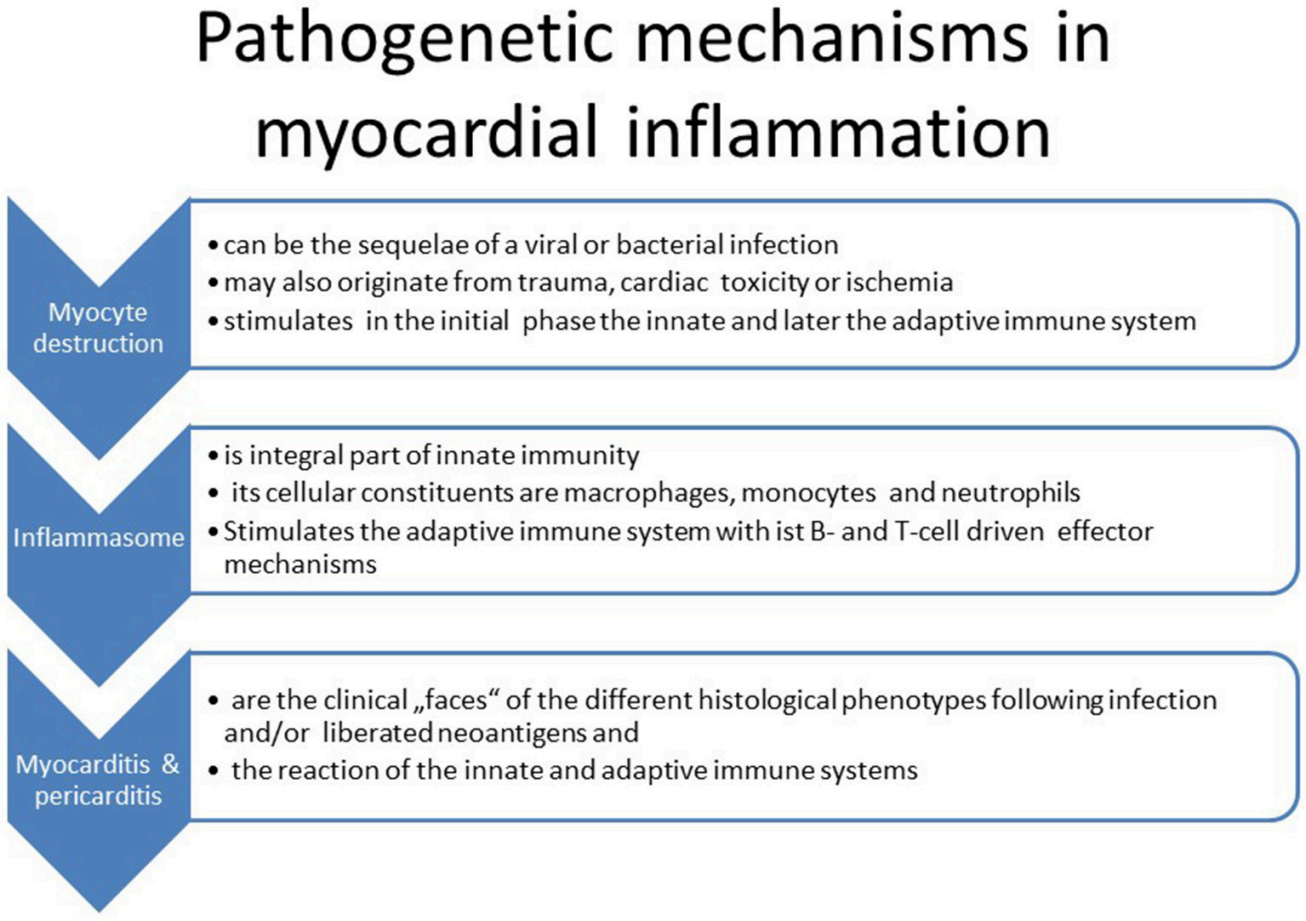
Sequence of events in myocardial inflammation.

11) Our understanding of the pathogenetic processes following viral infection or myocyte destruction has been widened particularly by analyzing the steps leading to the activation of the innate immune system. The innate immune system is triggered by pathogen-associated molecular patterns (PAMP) and damage-associated molecular patterns (DAMP via Toll-like (TLR) and Nod-like receptors (NLR). These receptors are assembled in the inflammasome, which is a multiprotein intracellular complex located predominantly in macrophages. It activates proinflammatory cytokines such as interleukin-1b and IL-18 after detecting infective microbial agents or sterile stressors. Inflammasomes can also induce pyroptosis, a form of programmed cell death. They can induce the adaptive immune system consequently (27). A dysregulation of inflammasomes can be associated with autoimmune syndromes such as an autoreactive myocarditis (Figure 2).

## Clinical Diagnosis in Human Myocarditis

According to the position statement of the European Society of Cardiology Working Group on Myocardial and Pericardial Diseases (8) the appropriate clinical work-up includes careful patient assessment for symptoms, auscultation, EKG for a new left bundle branch block (LBB) or severe recent rhythm disturbances at rest or exercise. Laboratory investigations should include cardiac biomarkers such as nt-pro BNP for the assessment of heart failure, troponin I or T for myocyte necrosis and c-reactive protein (CRP) for inflammation. Among the non-invasive imaging techniques echocardiography can indicate cardiac inflammation, when in a symptomatic patient exhibits segmental or global wall motion abnormality and coronary artery disease or left bundle branch block (LBB) are not present. A small pericardial effusion in this context may also lead the way. Cardiac MRI is very helpful by establishing cardiac inflammation or postmyocarditic lesions in a follow-up investigation by early or late gadolinium enhancement (LGE). In principle the Lake Louise MRI protocol should be followed. But no noninvasive diagnostic method can substitute endomyocardial biopsy to reach a final aetiological diagnosis, when histology, immunhistology and PCR for microbial agents are evaluated together.

Clinical symptoms can be diverse, from to life-threating cardiogenic shock and lethal ventricular rhythm disturbances, acute or chronic heart failure or an acute chest wall syndrome. In some cases they might even allow a suspicion of the underlying pathogenetic process (Table 1).

**Table 1:** From symptoms to aetiological diagnoses.

**Clinical phenotype**	**Symptoms and features**	**Aetiological diagnoses**
Acute life-threating heart failure, severe rhythm disturbance	Shock, NYHA III-IV, elevated Troponin I/T, elevated Nt-proBNP	Fulminant myocarditis, e.g., giant cell or eosinophilia or toxic myocarditis, borreliosis
Acute heart failure(AHF)	Dyspnoe, edema, reduce EF, but also diastolic AHF, variable EKG, intermittent Troponin I/T-and Nt-proBNP elevations	Viral or autoreactive myocarditis order inflammatory cardiomyopathy (DCMi)
Chronic heart failure(CHF)	CHF symptoms, no CAD, EKG, with LSB, RSB, AV-Block, variable ST-/T-alterations, some troponin I/T and Nt-proBNP elevations	Viral or autoreactive focal myocarditis or DCMi or borderline myocarditis
Acute chest wall syndrome	Angina like symptoms, but no CAD, variable ST-/T-alterations, in EKG, some Troponin I/T and Nt-proBNP elevations	Parvovirus B19 or other virus with or without pericarditis

Our understanding of the underlying aetiopathogenesis in man started some 35 years ago with the analysis of the antibody response to cardiac antigens in patients with suspected myocardial inflammation.

## Autoantibody—Mediated Immune Response in Human Myocarditis

**Figure 3:**
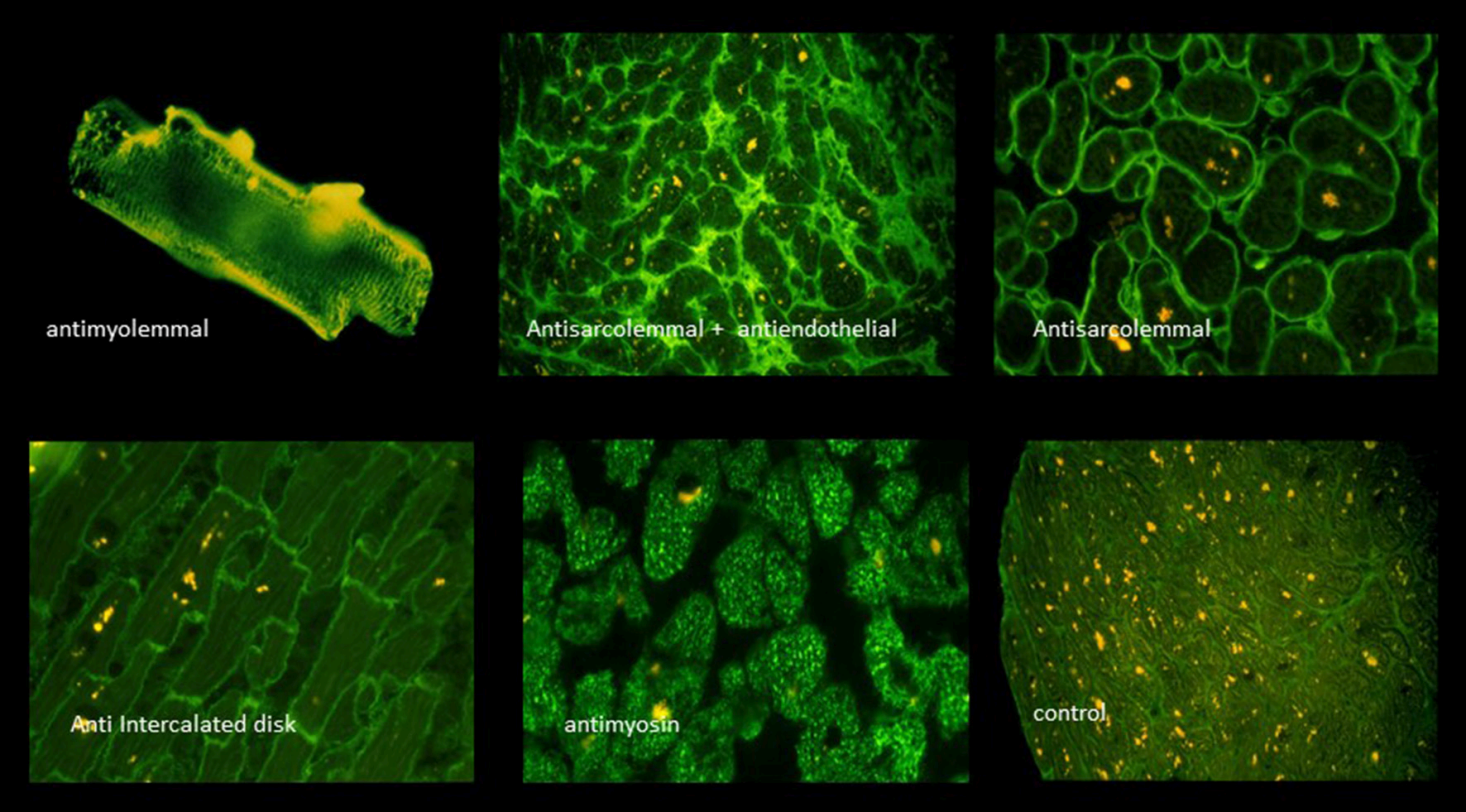
A selection of circulating anticardiac antibodies [from Maisch and Pankuweit (9) with permission from Springer-Nature].

**Figure 4:**
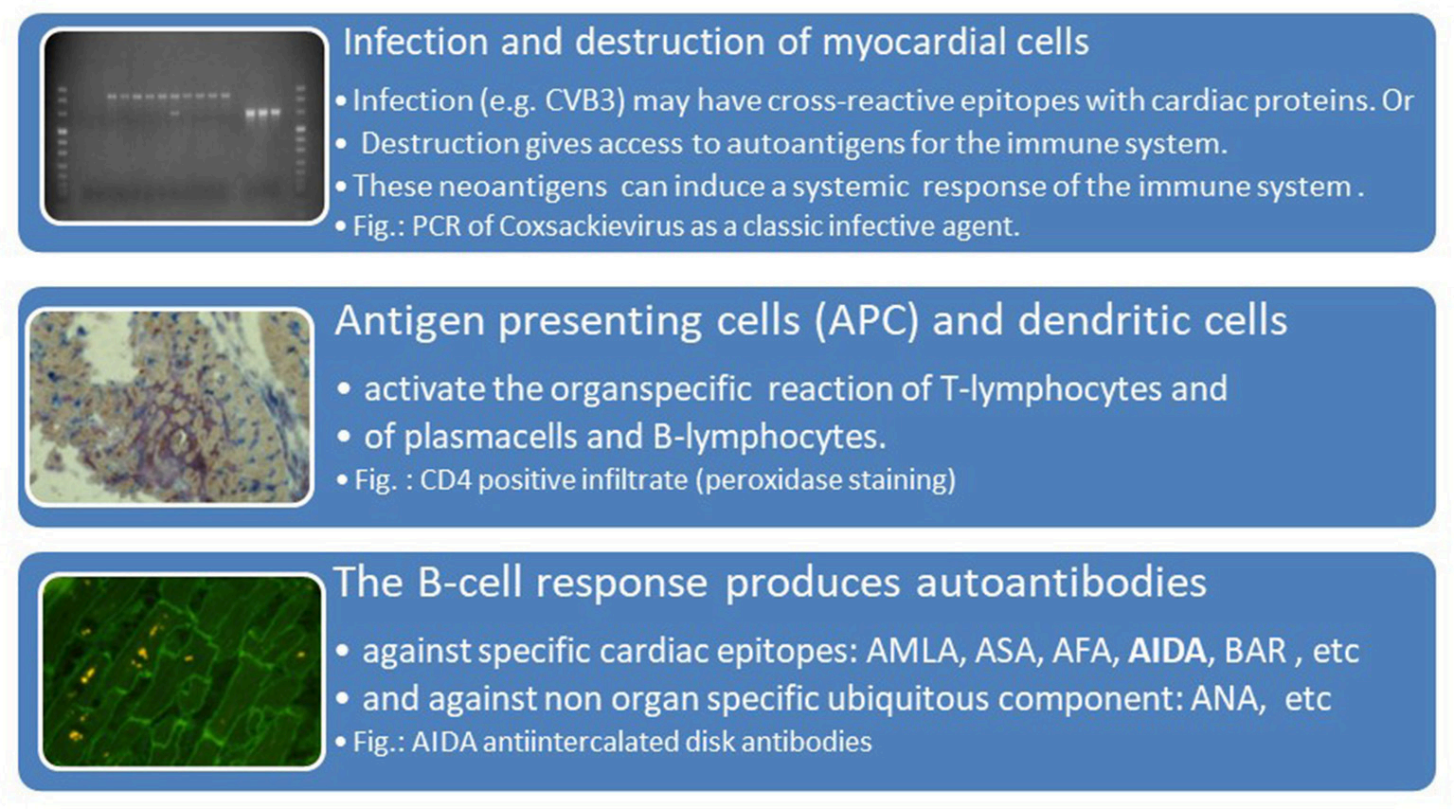
Viral infection, antigen presentation, response by the adaptive immune system [inserted images from Maisch and Pankuweit (9) with permission from Springer-Nature]. AMLA, antimyolemmal antibodies(ab); ASA, antisarcolemmal ab; AFA, antifibrillary ab; AIDA, antiintercalated disk ab(fig.); BAR, betarecepator ab; ANA, antinuclear ab.

The humoral immune response was at that time assessed by the indirect immunoflourescence test or Elisa against cardiac proteins together with testing for antibodies against cardiotropic viruses. At that time we have focused on antibodies cross-reacting between enteroviral epitopes with cardiac myolemma and sarcolemma (28–31). We also examined the prevalence and possible pathogenicity against laminin (32), fibrils, intermediate filaments (33), and against mitochondria (34, 35). Antibodies directed against myofibrillar proteins (36), troponins were in the focus of other investigators (37). Of particular interest was also the antibody response to the beta-receptor in the sera of patients with myocarditis and dilated cardiomyopathy (38, 39) and the muscarinic acetylcholine receptor (40). Of note, also the cardiac conduction tissue was addressed by the humoral immune response to the sinus and atrioventricular nodes and Purkinje fibers (41, 42) (Figures 3, 4, Table 2).

**Table 2:** Anticardiac antibodies [modified from Maisch and Pankuweit (9) with permission from Springer-Nature].

**Antigen**	**Antibody**	**Cross-reactivity**	**Pathomechanism**	**References**
Actin	Anti-actin	Unknown	Unknown	(43)
Acetylcholin-receptor	Anti-Ach	Unknown	Bradycardia	(44)
Aconitate hydratase	Anti-AH,	Unknown	Impaired metabolism	(31)
Adenin nucleotide translocator	Anti-ANT	Enterovirus	Impaired metabolism	(45)
Beta1-receptor	Anti-β1	Enterovirus	Pos. chronotropic	(38)
Beta1-receptor	Anti-β1		Neg. inotropic	(46, 47)
Creatine kinase	Anti-CK	Unknown	Impaired metabolism	(31)
Conduction system	Anti-sinus node Anti-AV node Anti-Purkinje	Unknown	Bradycardia AV-Block Conduction defect	(41, 42)
Desmin	Anti-desmin	Unknown	Unknown	(33)
Dihydrolipoamide dehydrogenase	Anti-DLD	Unknown	Impaired metabolism	(31)
Extracted Nuclear Antigens (ENA, SSA, SSB)	ENA, ANCA Anti-SSA Anti-SSB	Unknown	Neutrophil degranulation, Congenital AV-Block	(48)
Hsp60, hsp70, Vimentin	Anti-hsp60, Anti-hsp70, Anti-vimentin	Multiple	Unknown	(4)
Laminin	Anti-laminin	Unknown	Unknown	(32, 49)
Mitochondria /Microsomes	AMA	Multiple	Inhibition of sarcosin dehydrogenase	(31, 34, 50)
Myolemma	AMLA	Enterovirus	Lytic ab	(2, 28, 29)
Myosin	Anti-myosin	Enterovirus	Neg. inotropic	(51, 52)
Nicotinamideadenine-dinucleotide dehydro-genase	Anti-NADD	Unknown	Impaired metabolism	(31)
Nuclear Antigens	ANA	Unknown	Immune complex – mediated	(4)
Pyruvate kinase	Anti-PK	Unknown	Impaired metabolism	(31)
Troponin I (& T)	Anti-Troponin I	Unknown	Negative inotropic	(53)
Ubiquinol-cyto-chrome-c-reductase	Anti-UCR	Unknown	Impaired metabolism	(31)
Sarcolemma	ASA	Enterovirus	Lytic	(3, 24, 26)

*Ab, antibody; ASA, antisarcolemmal antibody; AMA, antimytochondrial antibody; AMLA, antimyolemmal antibody; Ach, acetylcholin; ANT, adenine nucleotide translocator; AH, aconitate hydratase; PK, pyruvate kinase; DLD, dihydrolipoamide dehydrogenase; CK, creatine kinase; NADD, nicotinamideadeninedinucleotide dehydrogenase; UCR, ubiquinol-cytochrome-c reductase; hsp, heat shock protein; ANA, antinuclear antigen; ANCA, anti-neutrophil cytoplasmic antigen; SR-Ca-ATPase, sarcoplasmatic reticulum calcium ATP-ase*.

## Immune Complexes

Since anticardiac antibodies may find their corresponding targets in the circulating blood, in serous body fluids or the targeted tissue itself immune complexes may also play a role in the pathogenetic process (54).

The most important question still is, which of these antibodies were only diagnostic markers of former myocyte destruction similar to the antibodies after a vaccination or which antibodies are pathogenetically truly harmful. Pathogenetic relevance was therefore attributed to those antibodies which were fixed to autologous cardiac tissue in the endomyocardial biopsy sample *in vivo* und proved cytolytic or protective in *in vitro* assays.

## Improvements in Histology, Immunohistology and Molecular Biology Methods

Endomyocardial biopsy (EMB) is the appropriate standard to diagnose myocarditis. It should be performed early in the course of the disease to optimize diagnostic accuracy and reduce the sampling error especially in focal myocarditis. Standard histology and immunohistology can be characteristic for certain types of inflammation (e.g., giant cell, eosinophilic myocarditis, sarcoidosis, lymphocytic). Immunohistology confirms the pathogenetic relevance of the autoantibodies, when they are fixed to the appropriate cardiac target protein. Polymerase chain reaction (PCR) identifies the underlying viral etiology or excludes it. This implies different treatment algorithms (13–17, 43, 55–59). Therefore, multiple specimens should be taken and immediately fixed in 10% buffered formalin. Additional samples should be snap frozen in liquid nitrogen for immunohistochemistry and stored at −80°C. And 1–2 samples should be stored in special tubes at room temperature for viral PCR (8, 15, 17, 60). To increase the diagnostic sensitivity of immunohistochemistry, the use of a large panel of monoclonal and polyclonal antibodies including anti-CD3, anti-CD4, anti-CD8, anti-CD68, and anti HLA-DR is mandatory for the identification and characterization of the inflammatory infiltrate (8, 15, 17). Quantitative immunohistochemistry should be performed for infiltrating cells. Specific binding of these antibodies indicating an inflammatory reaction is demonstrated by peroxidase double staining procedure.

Inflammation in endomyocardial biopsies is diagnosed by the WHF-criteria, which means a presence of ≥14 leucocytes/mm^2^ (8, 15) in European centers, whereas the qualitative Dallas criteria of active or borderline myocarditis in the first, and ongoing or resolving or resolved myocarditis in a subsequent biopsy are still applied in many American publications (Table 3).

**Table 3:** Comparison of qualitative Dallas (14) and quantitative World Heart Federation (WHF) criteria (15, 16).

**Biopsy diagnosis**	**Dallas criteria**	WHF criteria histology*	**WHF criteria viral etiology**
**1**^**st**^ **EMB:** Active myocarditis	Infiltrate (>5 per hpf or nests), myocytolysis, edema (only H&E staining)	>50/mm^2^ = fulminant m. >14/mm^2^ = active m.	(Quantitative) PCR on viruses If positive: viral m. or DCMi; If negative: autoreactive m.
Borderline myocarditis	Infiltrate (>5 per hpf or nests), (only H&E staining)	Not applicable	Not applicable
No myocarditis	No infiltrate	< 14/mm^2^	If negative: DCM If positive: viral DCM
**2**^**nd**^ **EMB:** Ongoing myocarditis	Infiltrate (>5 per hpf or nests), myocytolysis, edema (only H&E staining)	>14/mm^2^	(Quantitative) PCR on viruses, If positive: viral m. or DCMi; If negative: autoreactive m.
Healed/resolved myocarditis	No infiltrate, but focal fibrosis	< 14/mm^2^	If negative: DCM If positive: viral DCM

An equally important diagnostic contribution of EMB comes from the molecular analysis with DNA–RNA extraction and RT-PCR amplification of suspected viral genomes (15–17, 61), which is also part of the WHF criteria (Table 3). In order to exclude systemic infection, peripheral blood should be investigated in parallel with the biopsies (15, 17). Quantification of virus load and determination of virus replication may add diagnostic value (61). For detection of cardiotropic viruses total DNA and RNA should be extracted from the biopsy samples. Primer pairs specific for Coxsackievirus B (CVB), parvovirus B19 (PVB19), cytomegalovirus (CMV), adenovirus type 2, influenza virus A, human herpes virus 6 (HHV6) and Epstein–Barr virus (EBV) should be used to perform polymerase chain reaction (PCR) and in case of PVB19 quantitative real-time PCR to determine viral load.

## Histological Phenotypes and Clinical Manifestations

**Figure 5:**
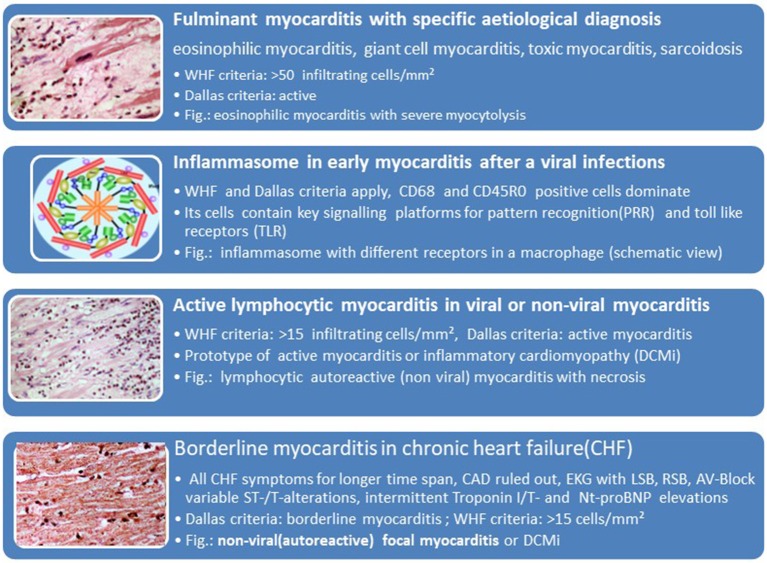
Histological phenotypes and components of myocarditis and inflammatory cardiomyopathy can correlate with clinical manifestations (faces).

The association of the clinical phenotypes such as cardiogenic shock with fulminant myocarditis, acute heart failure with active lymphocytic or other forms of viral and non-viral myocarditis and chronic heart failure with borderline myocarditis can be derived from Table 1 and Figure 5. The biopsy findings in these patients fit into these histopathological categories. The inflammasome is a platform in cells of the innate immune system allowing transition from the innate to the adaptive anticardiac immune response directed against myocardial and pericardial targets (11, 46, 62). The proinflammatory cascade in inflammasomes can be terminated intrinsicly, for example by Caspase-1 self-cleavage (63).

## Epidemiological Insight by Histology and PCR Based ETIOLOGIES in Patients With Suspected Myocarditis

**Figure 6:**
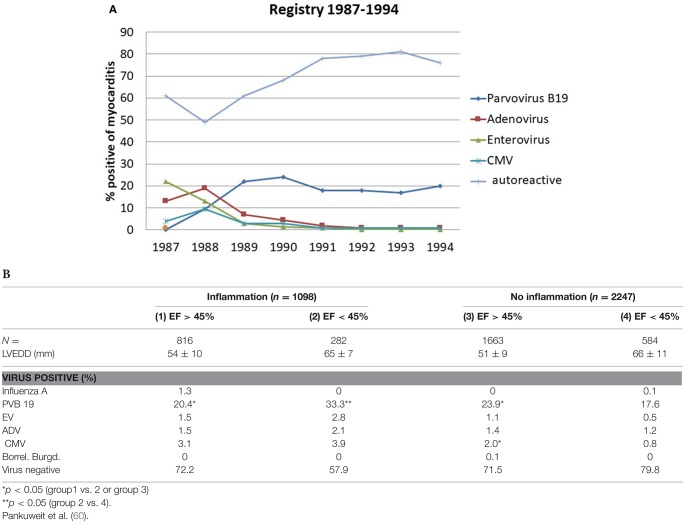
**(A)** Epidemiological shift in the Myocarditis Registry from entero-(green line) and adenoviruses (brown line) to Parvovirus B 19 (dark blue line) in the late 1980-ties. The number of patients with nonviral myocarditis (light blue line) varied from 50 to 80% in the same time span. **(B)** PCR-based etiology of viral and autoreactive myocarditis and dilated cardiomyopathy without inflammation (4th column) [modified from Pankuweit et al. (60)]. PCR-Based etiology of myocarditis and dilated cardiomyopathy. Investigation of endomyocardial biopsies from 3345 patients (1997–2003).

The last WHO report on the epidemiology of inflammatory heart diseases, which explicitly listed viral causes of myocarditis dates back to the year 1981. It filed the following incidences of viral myocarditis per 1000: Coxsackie- B 36, Influenza- B 18, Influenza A- 12, Coxsackie A- 10, Cytomegalo- 9, Echo- 7, Adeno- 5 und Epstein Barr Virus 4,5. Meanwhile epidemiological data show a wide divergence in different parts of the world with new endemics or epidemics. To follow epidemiological trends one can assess the incidence of aetiological factors in changing frequencies as assessed by endomyocardial biopsies in tertial referral centers. Their data have limits by the sample size of all biopsied patients per year as the denominator. The region or sometimes the continent could be another selection bias. Registries can show longitudinal trends, however. Our registry of suspected myocarditis / inflammatory dilated cardiomyopathy was started 1987. The histological diagnosis of myocarditis was based on the Dallas criteria in the first years 10 years. Later on we used the quantitative WHF-criteria and refined the PCR for cardiotropic microbial agents as part of a common consensus. By a longitudinal comparison an epidemiological shift from entero- and adenoviruses to Parvovirus B19 becomes apparent (Figure 6A).

A retrospective analysis of 3,345 patients‘ biopsies in the Marburg registry (60) revealed (Figure 6B):

- Only one third of the patients who underwent endomyocardial biopsy with the suspected diagnosis myocarditis or dilated cardiomyopathy showed inflammation in their biopsy.
- The greatest proportion of the patients was virus-negative, however. This applied to the patients with dilated cardiomyopathy with no inflammation. Such patients with an EF between 45 and 55% (3rd column) made up 71.5%, those with an EF <45% were virus-negative in 79.8%. These groups are identical with heart failure of unknown origin.
- Parvovirus B 19 became by far the leading viral aetiological factor across all 4 groups of cardiomyopathies with or without inflammation. It ranged between 17.6% in non-inflammatory but viral cardiomyopathy (= viral heart disease) and 33.3% in inflammatory Parvovirus B19 positive cardiomyopathy with an EF <45%.

## Future Serologic Diagnostic Markers

Distinct patterns of microRNAs are well described in coronary artery disease and myocardial infarction but not yet in inflammatory cardiomyopathies. De Rosa et al recently showed different gradients of microRNA expression in ischemic and non-ischemic forms of heart failure (64).

### Treatment

Current recommendations and guidelines for the treatment of heart failure also apply to inflammatory cardiomyopathy. “Unloading the heart” is the principle of chronic heart failure of any cause. This has been successfully demonstrated in many heart failure trials on ACE-inhibition and angiotensin receptor blockade. Details and a comprehensive bibliography were summarized previously (9). Waagstein et al. demonstrated first a positive trend for betablockade in congestive cardiomyopathy (65).

**Figure 7:**
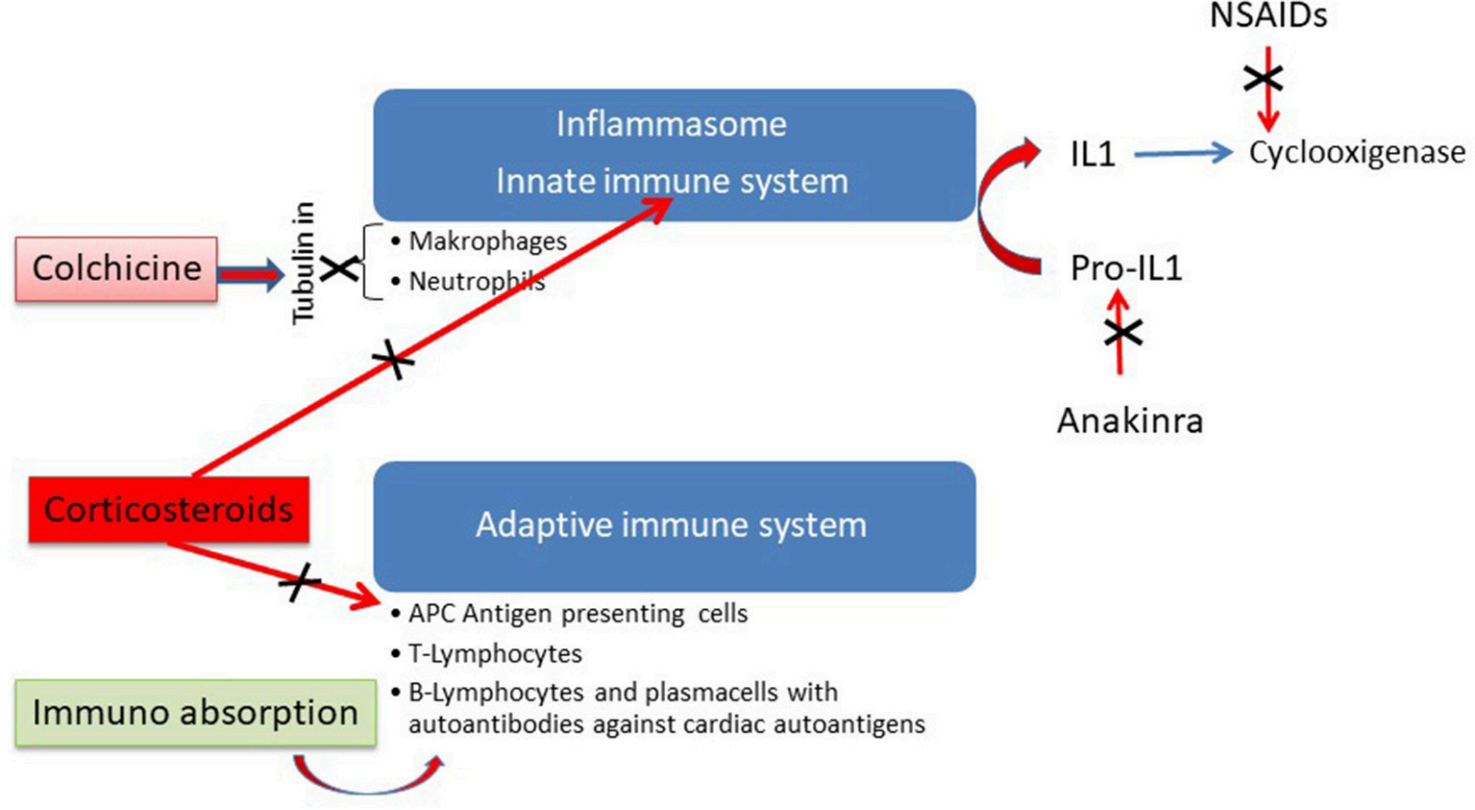
Antininflammatory action of different modes of therapy in perimyocarditis [modified from Maisch (69) with permission from Springer-Nature].

Antiphlogistic treatment with non-steroidal anti-inflammatory drugs (NSAIDs) such as ibuprofen or indomethacin or IL-antagonists such as anakinra should be reserved for patients with additional pericardial involvement. NSAIDs should be used only for short term application (66, 67), since in murine Coxsackie B3 myocarditis it was shown that NSAIDs can be detrimental (68). NSAIDs are cyclooxigenase inhibitors. Anakinra blocks the cytokine activity of IL-1. In peri (myo)carditis first line treatment with colchicine is now recommended not only in recurrent forms but also for the first attack of pericarditis (67). Figure 7 shows that colchicine is an inhibitor of the mitosis of tubulin in macrophages and neutrophiles. Its application inhibits primarily the innate immune system.

Antiarrhythmic treatment and device therapy also follows current heart failure guidelines [see Maisch and Pankuweit (9) for references].

### Stem Cell Transplantation

Virtually no data on stem cell transplantation in myocarditis with heart failure and only scarce uncontrolled data in patients with dilated cardiomyopathy are available (9).

## Specific Treatment Algorithms

**Figure 8:**
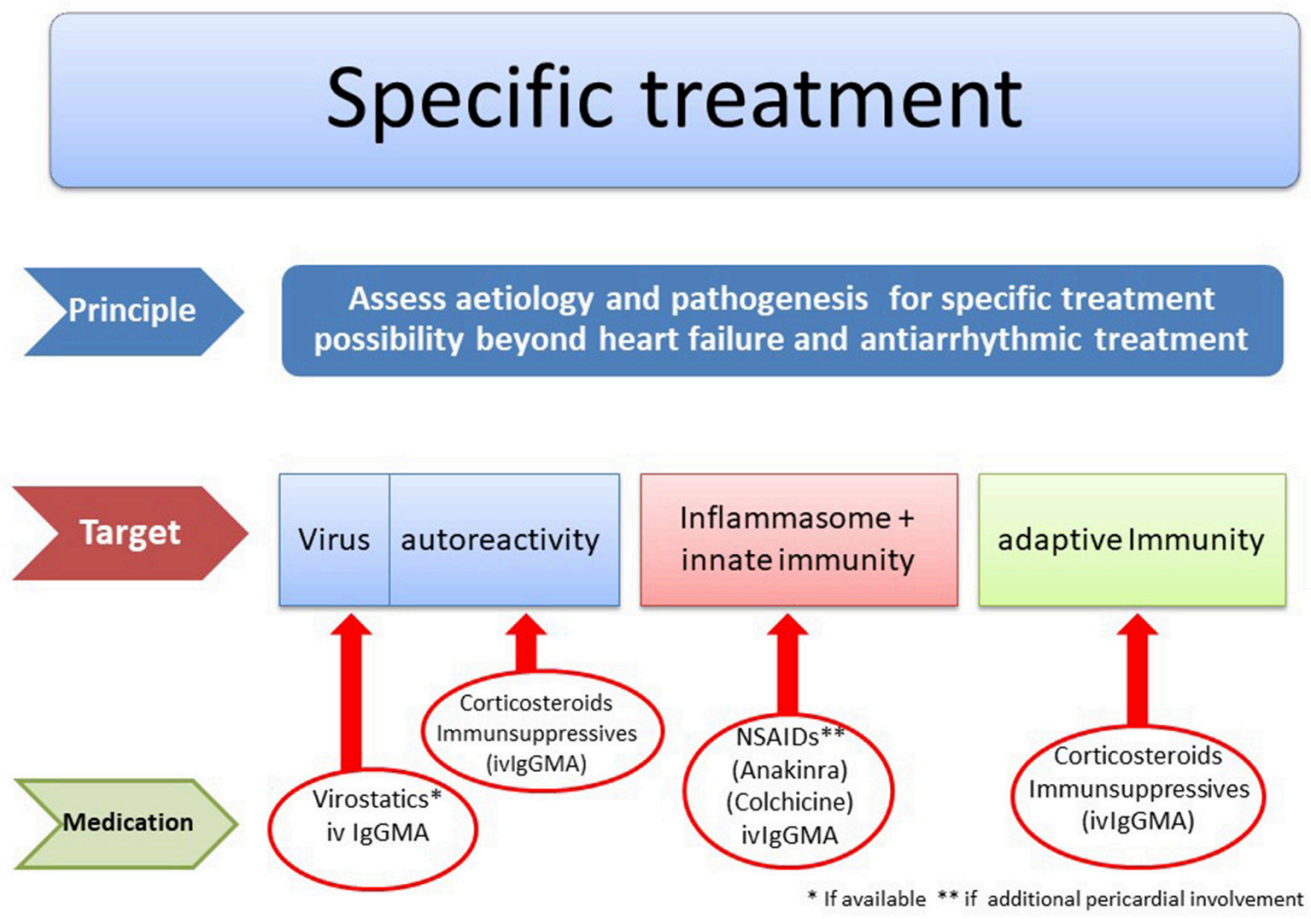
Etiology driven treatment in myocarditis and inflammatory cardiomyopathy [modified from Maisch (69) with permission from Springer-Nature].

Figure 8 reflects diagnostic and therapeutic algorithms in different forms of myocarditis. They are also the basis of the double-blind randomized European Study on Epidemiology and Treatment of Inflammatory Myocardial Disease (ESETCID) (70).

### Immunosuppressive Treatment

#### Idiopathic Giant Cell Myocarditis

Giant cells in addition to a lymphocytic infiltrate are the histological hall-mark of this very rare, fulminant and often lethal disease. If suspected, it is a clear biopsy indication. Its prevalence in Marburg registry 1989–2012 is 3 in 10,000 biopsied patients. The etiology is considered autoimmune based on a genetic predisposition. It resembles experimental giant cell myocarditis in Lewis rats after immunization with myosin (71). When compared to an isolated cardiac sarcoid the histological differential diagnosis is sometimes difficult (72). If untreated the natural course is lethal. The giant cell myocarditis treatment trial proposed treatment with 5 mg monoclonal anti CD3–antibodies given i.v. for 10 days. Cyclosporin should be started with 25 mg bid and increased daily by 25 mg to achieve a target serum level of 200 ng/ml. This serum level should be kept for 1 year. Methylprednisolone should be started with 10 mg/kg i.v. for 3 days and then be tapered after 3 weeks to a final dose of 5 mg for the rest of the year (73, 74). The study was stopped for lack of patients (Table 4).

**Table 4:** Trials for immunosuppressive treatment in myocarditis [modified from Maisch and Pankuweit (9) with permission from Springer-Nature].

**References**	**Treatment**	**No pts/Controls**	**Treated pts improved**	**Treated pts Unch/deter**.	**Controls improved**	**Controls unch/deter**.	**Endpoint/Comments**		
**A. OBSERVATIONAL STUDIES AND SMALL TRIALS WITH IMMUNOSUPPRESSION**								
Fenoglio et al. (75)	P, A & P	18/4	7 (39%)	11 (61%)	2 (50%)	2 (50%)	EF/observational, no PCR	
Hosenpud et al. (76)	A & P	6/0	0 (0%)	6 (100%)			EF/No co, no PCR, no co biopsy	
Anderson et al. (77)	A & P	10/7	3 (30%)	7 (70%)	2 (28,5%)	5 (71,5%)	Prospective, open label, randomized	
Marboe and Fenoglio (78)	P, A & P	16/18	9 (56%)	7 (44%)	7 (39%)	11 (61%)	P, A & P mixed	
Latham et al., (79)	P	26/26	Majority	Minority	nd	nd	EF/No viral PCR, no biopsy	
Maisch et al. (80)	A & P	21/21 all virus negative	10 (47%)	11 (53%)	3 (14%)	18 (86%)	EF (6 mo)/RCT pilot	
Kühl et al. (81)	P	31/0	20 (54%)	11 (46%)	nd	nd	EF; observational/ No co EMB	
Camargo et al. (82)	P	68/0	Majority	Minority	nd	nd	EF/observational, No viral PCR	
Liu Dezue et al. (83)	D	128/0	Favorable, but no data	nd	nd	nd	Observational/No EMB, CM	
Sun (84)	D	32/0	Majority	Minority	nd	nd	EF/observational, EKG only, no PCR, CM	
Wu and Chen (85)	D & P	31/0	Majority	Minority	nd	nd	Observational/ No EMB, CM	
Frustaci et al. (86)	A & P	41/0	21 (51%)	20 (49%)	nd	nd	EF/RCT, virus negative pts improved	
Escher et al. (87)	A & P	114	Majority	Minority	nd	nd	EF 6 mo/observational, no co biopsy	
**References**	**No pts/Co**	**Treatment**	**Endpoints**	**Treated pts improved**	**Treated pts Unch/deter**.	**Controls improved**	**Controls unch/deter**.	**Comments**
**B. DOUBLE BLIND, RANDOMIZED, AND CONTROLLED TREATMENT TRIALS (RCT) WITH IMMUNOSUPPRESSIVE DRUGS IN MYOCARDITIS**								
Parillo et al. (88)	51 /51	P vs. Pl	EF after 3 mo, mortality	53% No difference	47% No difference	27% No difference	73% No difference	RCT, no PCR
Mason et al. (89) MTT	64/47	A/C & P vs. Pl	EF/function +Mortality	No difference No difference	No difference No difference	No difference No difference	No difference No difference	RCT, no PCR (90)
Wojnicz et al. (91)	41/43	A & P vs. Pl	EF/function	In majority	In minority	Minority with spontaneous improvement	Majority	No PCR, HLA as criterium of inflammation
Cooper et al. (74), Maisch et al. (69, 70)	11/?	Cyclo+P vs. Pl	Mortality 12 mo	Improved	nd	nd	nd	RCT, stopped for lack of pts
Frustaci et al. (92) TIMIC	43/42	A & P vs. Pl	EF (6 mo) Mortality	88,3 nd	11,7 nd	0 nd	100 nd	WHF, RCT, virus negative pts only
Maisch et al. (93) ESETCID	54/47	Tx arms with A& P vs. Pl	EF/function MACE	EF+MACE improved after 6 month		Some spontaneous improvement		WHF, RCT, intermediate results

*A, Azathioprin; co, controls; CM, Chinese medicine; Cyclo, Cyclosporine; D, Dexamethason; DC, dilated cardiomyopathy; deter, deteriorated; EF, Ejection fraction; EMB, endomyocardial biopsy; mo, months; nd, no data; P, prednisone; Pl, placebo; PCR, polymerase chain reaction for viral RNA and DNA in EMB; pts, patients; RCT, randomized controlled trial; unch, unchanged; WHF, quantitative World Heart Federation biopsy criteria*.

#### Cardiac Sarcoidosis

In cardiac sarcoidosis the giant cells are only found in the non caseous granuloma (94, 95). In our registry it is 6-times more frequent with 19 in 10,000 biopsied patients than giant cell myocarditis. Granuloma are often located in the midmyocardial layer, which is not accessible to endomyocardial biopsy. So the diagnosis of cardiac sarcoidosis can be suspected in patients with systemic sarcoidosis and a biopsy just showing myocarditis. Early cardiac symptoms can be AV-block or severe ventricular arrhythmias leading to sudden death or severe heart failure. The etiology of sarcoidosis remains obscure, although recently a variant in the *btnl2* gene and the *btnl2* risk allele were described as risk factors (9, 96, 97). The treatment is either corticoid treatment alone or in combination with other immunosuppressive drugs e.g., azathioprine or cyclosporine (98).

#### Eosinophilic Heart Disease

Eosinophilic heart disease (EHD) and endstage endomyocardial fibrosis are rare diseases. The Marburg Registry has collected 10 cases over 23 years. Its common pathogenetic denominator is the excessive production of cytotoxic eosinophils, which could damage the heart in different ways (9, 99, 100):

- a) In the course of an allergic reaction,
- b) As an autoimmune disease,
- c) As malignant eosinophilic leukemia,
- d) Following a parasitic or protozoal infection (tropical form),
- e) As Churg-Strauss-syndrome or
- f) As idiopathic form.

Classic Löffler's endocarditis develops in 3 stages:

1. *Eosinophilic endomyocarditis*, in which mature eosinophils infiltrate the endocardium and myocardium and damage with their products such as the cationic protein or by IL-5, which has also been discussed as a late mediator of fibrosis.
2. *Thrombotic endocardial disease*, in which apical obliteration and valve involvement occur.
3. *Endomyocardial fibrosis* as terminal stage, in which restrictive cardiomyopathy prevails.

The 3 stages can be identified noninvasively by colorflow Doppler echocardiography, cardiac MRI, and by EMB. In the peripheral blood the eosinophils can sometimes be degranulated. They are diagnosed as neutrophils, which obviously impairs the diagnosis of eosinophilia. The definite diagnosis should be established by endomyocardial biopsy. The causative therapy of the tropical form is the treatment of the underlying helminthic or protozoal infection. In all other forms immunosuppression has been recommended either by prednisone, interferon or the tyrosinekinase inhibitors imatinib or mepolizumab. As a humanized monoclonal antibody mepolizumab binds to and inhibits interleukin-5 (IL-5). In the Marburg registry longterm prednisone and azathioprine gave a survival rate of 9 out 10 patients over a mean period of 8.4 years (9).

### Rheumatic Diseases and Collagen Disorders With Cardiac Manifestations

Cardiac symptoms may be “behind the curtain” of the clinical manifestations in rheumatic diseases. The diagnosis relies on clinical manifestation, echocardiography, cardiac MRI, and sometimes on endomyocardial biopsy and/or pericardiocentesis. The management includes pain relief with NSAIDs, immunosuppression as systemic therapy, and in patients with larger pericardial effusions undergoing pericardiocentesis with intrapericardial instillation of triamcinolone acetate. Longterm, oral colchicine (2–3 tablets per day with 0.5mg) is recommended (9).

### Autoreactive Myocarditis

It is common belief that an infection with cardiotropic viruses may cause sequestration of myocardial cells. This can trigger in predisposed patients an autoreactive cellular and humoral immune reaction which in turn leads to further myocardial damage.

With this hypothesis in mind immunosuppressive treatment either by prednisone alone or in combination with azathioprin or cyclosporin was initiated. However, most studies listed in Table 4A were carried out before quantitative immunohistochemistry for the assessment of the infiltrate and PCR for cardiotropic viruses were available. So it remained unclear, if prednisone and immunosuppression were started when virus particles were still present. According to a current dogma, in such a situation immunosuppression is contraindicated.

Our controlled pilot study on immunosuppression (80) before the initiation of ESETCID (93) excluded patients with a viral genome in the myocardium and was therefore directed to autoreactive, virus-negative myocarditis cases. It demonstrated improvement of cardiac function (EF >5% after 6 months) in 47% of patients treated with verum, but also in 14% in the placebo group, which could be interpreted as spontaneous recovery.

In a *post-hoc* stratification of myocarditis patients treated with prednisone and azathioprine Frustaci et al. (86) also found that improvement with immunosuppression was demonstrable only in the virus-negative cases.

The first randomized controlled trial on prednisone in patients with idiopathic dilated cardiomyopathy with biopsies taken was carried out by Parillo et al (88), who randomly assigned 102 DCM patients to treatment with 60 mg/d for 3 months or without prednisone. 53% of the patients who received prednisone showed improvement of ejection fraction by >5%, but only 27% of the controls improved spontaneously (*p* = 0.005).

The Myocarditis Treatment Trial (MTT) by Mason et al. (89) showed neither benefit nor harm. Mortality after 6 months of treatment with cyclosporin A or azathioprine and prednisone showed an insignificant trend when compared to placebo. The study was underpowered and did not distinguish viral from non-viral disease as pointed out later (90). Wojnicz et al. (91) randomized 84 patients with dilated heart muscle disease and suspected myocarditis when an increased HLA MHC expression was found in EMB. Treatment of azathioprine and prednisone was compared with placebo after 3 months. In the treatment group ejection fraction improved, survival remained comparable, however, between verum and placebo groups.

In the TIMIC study the ejection fraction in the treatment group of 43 patients increased from 26.5% at baseline to 45.6% after 6 months (*p* < 0.001). Accordingly left ventricular enddiastolic volume, left ventricular enddiastolic diameter, and New York Heart Association class decreased significantly (92).

The ESETCID (European Study on the Epidemiology and Treatment of Cardiac Inflammatory Disease) is a double blind, randomized, placebo controlled three-armed trial with prednisolone and azathioprine for autoreactive (virus negative) inflammatory dilated cardiomyopathy in patients with an ejection fraction <45% at baseline. Its intermediate results from the immunosuppressive treatment arm demonstrated a positive trend in EF and MACE after 6 months of treatment and a significant benefit after 1 year of follow-up (93). For the initial and steady state dosages of prednisolone and azthioprine see Maisch et al. (93). The control group without immunosuppressive treatment also showed some spontaneous resolution of the infiltrate.

## Intravenous Immunoglobulin

Intravenous immunoglobulins (ivIg) interact widely with the host immune system. They can stimulate anti-inflammatory cytokines, develop anti-idiotypic activities, increase FCgamma receptor saturation and the expression of the inhibitory FCgRIIB. Inhibitory actions comprise the suppression of proinflammatory cytokines, the interruption of the complement cascade, the inhibition of dendritic cells, of leukocyte adhesion, of apoptosis and of metalloproteinases. They can bind microbial particles, contribute to the self-antigen sequestration and interfere with B and T cell regulation (9). Anthony et al have shown that the anti-inflammatory activity of monomeric IgG depends on the sialysation of the N-linked glycan of the IgG Fc fragment (101). Their beneficial effect has also been reported in different clinical settings of autoimmune disease including acute and chronic myocarditis, in dilated cardiomyopathy, in experimental enteroviral myocarditis (102) and in Parvovirus B19 associated heart disease (103). IgM and IgA enriched immunoglobulins appear to be effective in lower doses (104).

Table 5 gives an overview on the ivIg studies. Many, but not all studies reported hemodynamic benefit or clinical improvement. The IMAC, a randomized controlled trial, demonstrated improvement in both, the treatment and placebo arms (110), so that in a multi-institutional analysis the benefit in a pediatric myocarditis or cardiomyopathy population was questioned (118).

**Table 5:** Iv-Immunoglobulin treatment in myocarditis and inflammatory dilated cardiomyopathy.

**References**	**Design**	***n***	**EMB**	**Viral PCR**	**IvIg dose**	**Outcome**
Drucker et al. (105)	Retrospective, historic control	46 children	Partly	nd	2 g/kg single dose	Reduced LVEDD
McNamara et al. (106)	Uncontrolled	10 adults	Partly	nd	2 g/kg	Improvement of EF after 12 months
Takeda et al. (107)	Case report	1	Myocarditis	EBV	2 g/kg for 2 days	Improvement
Nigro et al. (108)	Case reports	3 children	Myocarditis	Parvo B19	2 g/kg over 5 days	Improved
Tsai et al. (109)	Case report	1 child	nd	Mycoplasma peumoniae (serology)	2 g/kg over 2 days	Improved
McNamara et al. (110) IMAC	RCT	62	Only ten active and 3 borderline myocarditis	nd	2 g/kg, single shot vs. controls	Not improved
Alter et al. (111)	Case report	1	Myocarditis	Varicella	2 g/kg over 2 days	Normalized
Shioji et al. (112)	Case report	1	Fulminant myocarditis	nd, negative serology	2 g/kg	Improved
Tedeschi et al. (113)	Case report	1	nd	nd, negative serology	2 g/kg	Improved
Kishimoto et al. (114)	Case series	9 adults	4 myocarditis only	nd	1-2 g/kg	Improved NYHA, EF & SF
Wang et al. (115)	Case report	1 child	Fulminant myocarditis	Coxsackie A 16	1/kg for 2 days	Patient died
Dennert et al. (116)	Uncontrolled	25	post mortem myocarditis	Parvo B19	2 g/kg	Decrease in viral load and improved EF after 6 months
Maisch et al. (117)	Controlled	18 (ivIg) vs. 17(controls)	CMV myocarditis	CMV by PCR or ISH	14 days, multiple doses	Improved and eradicated CMV

*Modified from Maisch and Pankuweit (9, 10) with permission from Springer-Nature*.

*CMV, Cytomegalovirus; DC, dilated cardiomyopathy; deter, deteriorated; EBV, Epstein Barr virus; EF, Ejection fraction; EMB, endomyocardial biopsy; ISH, in situ hybridization; LVEDD, left ventricular enddiastolic diameter; NYHA, New York Heart Association classification; mo, months; n, number of pts; nd, no data; PCR, polymerase chain reaction for viral RNA and DNA in EMB; RCT, randomized controlled trial; SF, shortening fraction; unch, unchanged*.

In CMV-myocarditis one controlled trial of 18 patients showed the eradication of inflammation and the elimination of the virus (117). The patients had received 2 ml/kg i.v. cytomegalovirus hyperimmunoglobulin (CMVhIg) for 3 days and 1 ml/kg for 2 additional 2 days.

In a case of varicella myocarditis high-dose immunoglobulins demonstrated clinical improvement and the resolution of inflammation (111).

In the Marburg Registry 20 g i.v. pentaglobin (ivIgGAM) given in adenoviral myocarditis resulted in clinical improvement by the eradication of the inflammatory infiltrate and the virus. In Parvo B19 myocarditis clinical improvement and elimination of inflammation in the biopsy is noted, whereas the virus may still persist although the viral load may decrease.

## Immunoadsorption

The therapeutical concept of immunoadsorption follows a different concept: the elimination of cardiotoxic autoantibodies together with proinflammatory cytokines. The positive result of a pilot study of patients with idiopathic dilated cardiomyopathy needs further confirmation in a larger endpoint study (119, 120).

## Antiviral Treatment

### Interferon-Beta

Interferons belong to the natural defense system against many viral infections. In entero- and adenoviral myocarditis interferon-beta has eliminated the viral genome and decreased inflammation in a phase 2 study, when applying dosages of 2 to 6 × 10^6^ IU every 2nd day (121). The response to interferon-beta in Parvovirus B19 and human herpes virus 6 myocarditis has been less impressive as shown by the BICC study (122).

## Perspective and Conclusion

Although we have learned much about inflammatory heart disease from various animal models of viral or autoimmune myocarditis, we are aware that animal models cannot be translated one to one to myocarditis in patients. Enteroviral myocarditis in man has almost completely disappeared in Europe. Parvovirus B 19 as infective agent has emerged instead but its pathogenesis is still poorly understood and animal models for this virus are still missing. Separation of myocarditis in 3 phases is an auxiliary construction. But myocardial inflammation in man is continuum. Personalized treatment should be tailored within the time frame from infection to innate and adaptive response. There is still much work to be done.

## Author Contributions

The author confirms being the sole contributor of this work and has approved it for publication.

### Conflict of Interest Statement

BM has received honoraria for lectures from Biotest Co.
